# The protocol for a multisite, double blind, randomized, placebo-controlled trial of axillary nerve stimulation for chronic shoulder pain

**DOI:** 10.1186/s13063-020-4174-x

**Published:** 2020-03-06

**Authors:** Travis Cleland, Nitin B. Jain, John Chae, Kristine M. Hansen, Terri Z. Hisel, Douglas D. Gunzler, Victoria C. Whitehair, Chong H. Kim, Richard D. Wilson

**Affiliations:** 1 MetroHealth Rehabilitation Institute, MetroHealth System, 4229 Pearl Rd, N5-27, Cleveland, OH 44109 USA; 2 Vanderbilt University Medical Center, 3319 West End Ave, Nashville, TN 37203 USA; 3 Center for Healthcare Research and Policy, MetroHealth System, 2500 MetroHealth Dr., Cleveland, OH 44109 USA

## Abstract

**Background:**

Shoulder impingement syndrome is one of the most common causes of shoulder pain, accounting for approximately 30% of all shoulder pain. Approximately 35% of patients with shoulder impingement syndrome are refractory to conservative treatment. For patients who fail conservative treatment, there is no established treatment to successfully treat their chronic pain. Prior randomized control trials have demonstrated efficacy for the use of a single lead intramuscular peripheral nerve stimulation of the axillary nerve at the motor points of the deltoid muscle for treatment of hemiplegic shoulder pain. This is the first controlled trial to utilize the same novel technology to treat shoulder impingement syndrome outside of the stroke population.

**Methods:**

This is a dual-site, placebo-controlled, double-blinded, randomized control trial. Participants will be randomized to two treatment groups. The intervention group will be treated with active peripheral nerve stimulation of the axillary nerve of the affected shoulder and the control group will be treated with sham peripheral nerve stimulation of the axillary nerve of the affected shoulder. Both groups will receive a standardized exercise therapy program directed by a licensed therapist.

**Discussion:**

This study protocol will allow the investigators to determine if this novel, non-pharmacologic treatment of shoulder pain can demonstrate the same benefit in musculoskeletal patients which has been previously demonstrated in the stroke population.

**Trial registration:**

Clinicaltrials.gov, NCT03752619. Registered on 26 November 2018.

## Background

Subacute and chronic shoulder pain accounts for nearly 12 million physician office visits and consumes approximately 7 billion dollars in direct costs annually [1]. The prevalence of shoulder pain is approximately 7%–27% in adults aged < 70 years. Shoulder impingement syndrome is one of the most common causes of shoulder pain, accounting for approximately 30% of all shoulder pain [2, 3]. Multiple shoulder pathologies co-exist within the clinical diagnosis of shoulder impingement syndrome, though the anatomic pathology of shoulder impingement syndrome refers to the supraspinatus tendon impinging on the under surface of the acromion with shoulder abduction [4]. Approximately 35% of patients with shoulder impingement syndrome are refractory to conservative treatment [5, 6]. As the duration of symptoms continues, the likelihood of success of treatment declines [7]. Many of these patients ultimately proceed to surgical treatment [5, 6, 8, 9]. Unfortunately, surgery has not been shown to be superior to conservative treatment [10–15]. Of all upper limb arthroscopic surgeries, 34% account for treatment of shoulder impingement syndrome [5, 6, 8, 9]. For patients who fail conservative treatment, there is no established treatment to successfully treat their chronic pain.

Prior randomized control trials (RCT) have demonstrated efficacy for the use of a single lead intramuscular peripheral nerve stimulation (PNS) of the axillary nerve at the motor points of the deltoid muscle for treatment of hemiplegic shoulder pain [16–18]. This is the first RCT to utilize the same novel technology to treat shoulder impingement syndrome outside of the stroke population. Unlike spinal cord stimulation or other surface stimulation modalities, PNS has demonstrated pain reduction for up to 12 months after termination of stimulation [7, 16, 19–22]. A case series of 10 participants supports the hypothesis that intramuscular PNS offers pain control of patients with shoulder impingement syndrome [23]. Prior investigators have concluded that an intramuscular stimulation system can elicit a strong muscle contraction without activating the skin nociceptive fibers, therefore leading to avoiding discomfort associated with surface stimulation [24–26].

There is evidence that chronic pain is linked to maladaptive neuroplasticity leading to central sensitization [27]. Central sensitization is described as an increase in activity in neurons and nociceptive circuits that enhances the sensation of pain. Multiple lines of research have shown that persons with chronic shoulder impingement syndrome demonstrate signs of central sensitization [28–32]. It is theorized that treatment aimed at reducing central sensitization will be effective in reducing chronic pain and that PNS may affect the central nervous system to reduce central sensitization [17, 18, 23, 33]. Prior studies have demonstrated neuroplasticity of the motor and sensory cortices after PNS that persists after stimulation has ceased [34–38]. Additionally, a meta-analysis concluded that PNS above the motor threshold causing muscle contractions may be superior to sensory stimulation at producing neuroplasticity at the cortical level [35]. The meta-analysis also concluded that this is dose-dependent, meaning the longer the duration of stimulation, the greater the cortical changes [35]. It is postulated that the golgi tendon organs and muscle spindles are activated by the repetitive muscle contraction causing an afferent signal to the central nervous system resulting in the neuroplasticity. This proprioceptive input into the central nervous system cannot be produced by TENS, peripheral nerve field stimulation, or even posterior spinal cord stimulation.

In prior studies assessing PNS for shoulder impingement syndrome and post-stroke shoulder pain, approximately 60% of participants demonstrated successful treatment of their chronic shoulder pain [16, 33]. The specific reason for why some participants respond and some do not is not fully understood and therefore needs further investigation.

## Methods

### Objectives

The first objective of this trial is to determine the efficacy of intramuscular PNS of the axillary nerve for treatment of chronic pain due to shoulder impingement syndrome. It is hypothesized that participants with intramuscular PNS of the axillary nerve will experience greater reduction in shoulder pain and impairment. The second objective is to further understand the relationship in pain reduction with the use of intramuscular PNS of the axillary nerve and changes in the somatosensory system that suggests an association with reduction in central sensitization. It is hypothesized that participants with pain reduction with intramuscular PNS will experience increased pain thresholds not only on the affected shoulder, but also in a generalized pattern throughout the body. The third objective is to determine if a specific phenotype of responders can be identified.

### Design

This is a dual-site, placebo-controlled, double-blinded RCT. Both sites, one in Ohio and one in Tennessee, are academic hospitals in the United States. This study protocol is institutionally review board approved.

### Ethical considerations

The coordinator or other authorized research team members review the HIPAA forms, Alternate Means of Communication forms, and consent forms with the participants. The coordinator and authorized research team members who obtain informed consent with participants are trained and approved by the Institutional Review Board. The potential participants are informed of the purpose and nature of the study, the potential hazards and risks, the potential benefit, the procedure, what is expected of the participants, and the expected follow-up after intervention. Participants are informed that participation is voluntary and that withdrawal from the study may occur at any time. Participants are asked if they agree for their data to be used should they choose to withdraw from the trial. Participants are also asked for permission for the research team to share relevant data with people from the Universities taking part in the research or from regulatory authorities, where relevant. This trial does not involve collecting biological specimens for storage.

### Inclusion criteria

Participants are those who demonstrate shoulder pain > 3 months, who are aged ≥ 21 years, with worst pain in the last week ≥ 4 on a numeric rating scale of 0–10, on a stable dose of no more than one pharmacologic analgesic, and with the ability to perform dressing changes and skin checks.

### Exclusion criteria

The study excludes participants who have current shoulder joint or overlying skin infection, current bacterial infection of any location on antibiotics, other chronic pain syndrome of another area of the body that requires consumption of analgesics for pain for at least 15 out of the past 30 days, prior shoulder surgery on the ipsilateral shoulder, corticosteroid injection to the ipsilateral shoulder within in the prior 12 weeks, uncontrolled bleeding disorder, medical instability, pregnancy, neurologic syndrome affecting the ipsilateral arm, current workers compensation claim on the ipsilateral arm, shoulder instability, history of severe trauma to the ipsilateral shoulder, assault to the ipsilateral shoulder, current osseous fracture of the ipsilateral shoulder, ipsilateral limb amputation other than a single digit, surgical indication for the ipsilateral shoulder, compromised immune system, current use of a deep brain stimulator, a tape or adhesive allergy, valvular heart disease at high risk for infective endocarditis, any implantable cardiac stimulator, and any other implantable neurostimulator. Participants are asked to refrain from other occupational therapies or physical therapies to improve shoulder pain, electrical stimulation to any other part of the body, other experimental procedures of the upper limb, ipsilateral shoulder injections, and any change in analgesic medications. Participants can use one pharmacologic analgesic, opioid or not. The participants can adjust the dose of the medication but are not to add analgesic medications during the protocol.

### Discontinuation criteria

Treatment would be discontinued based on the individual’s desire to stop participation or if there was evidence of an adverse outcome because of treatment that could only be corrected by stopping treatment.

### Recruitment

The targeted number of participants is 116. This is a dual-site study and each site employs strategies appropriate to recruit in their local environment. The study is listed on ClinicalTrials.gov (NCT03752619) with necessary contact information. Outpatient clinics are likely to be the largest generator for recruitment. The study coordinator identifies participants via multiple means. First, by the participant response to local advertisement, study flyer, or through the study website. Second, by the information on clinicaltrials.gov. Third, through medical record screening during outpatient visits at clinical sites. Fourth, through referral from physician, therapist, or other medical staff. Finally, through other means of community-based outreach. Each site has an approved HIPAA waiver for participant screening. Monthly meetings are held for sites to discuss recruitment plans and share information on recruitment strategies which offer the highest yield. The study uses a staged screening process to assist with identifying these individuals by pre-screening only participants by telephone and an in-person eligibility assessment for those who are more likely to meet the selection criteria. Financial remuneration for time spent in study activities includes a $15 compensation for completing each therapy visit and $50 compensation for completing each assessment visit. The total remuneration is $220 for completing the entire study protocol.

### Randomization, allocation concealment, and blinding

Randomization of the participants is completed by utilizing a secure online database. Once the participant is recruited, the randomization code will be released after all baseline assessments have been completed, allowing for concealment. The allocation sequester will be generated by a stratified block randomization scheme, which is housed within the secure online database. Participants are stratified based on baseline pain (> 6 or ≤ 6), duration of pain (> 18 months or ≤ 18 months), and study site [39–41].

The treatment group is known by the interventional physician at the time of the procedure and by the project coordinator. The treatment therapist may know the treatment group, depending on roles at each site. The assessing therapist and the participants are blinded to group assignment. To ensure the assessment therapist remains blinded, the treatment visits are scheduled at a location separate from assessment therapist. Additionally, the participants will be instructed to only discuss stimulation system issues with the treating therapist or study coordinator, not the blinded assessing therapist. Unblinding is not intended to occur. If this does occur, the staff will record the ID of the unblinded patient, the reason for unblinding, the staff involved in the unblinding incident, the staff who have been unblinded, and when the unblinding occurred during the participation. This will be recorded in the study database. All participants are queried at the conclusion of their participation to assess the success of blinding.

At baseline, the participants answer questionnaires regarding pain-related psychological traits, mood, demographics, co-morbidities, and medication use. All participants who do not have a contraindication to magnetic resonance imaging (MRI) of the shoulder will undergo an MRI scan of the ipsilateral shoulder, though an existing MRI scan can be used if it was obtained during the same episode of shoulder pain. The MRI is used to: document cystic or sclerotic changes to the greater tuberosity, sclerosis, or spurring of the acromion, arthritic changes to the acromioclavicular joint; assess tendons for tears, tendinosis, tendonitis, or calcifications; evaluate the glenohumeral joint for stability and arthritic changes; and assess the subacromial bursa for fluid [9].

### Outcome measures

The primary outcome measure is the worst pain at the affected shoulder within the last week measured by the Brief Pain Inventory (BPI). The BPI is recommended by the IMMPACT group for its outstanding psychometrics for the assessment of pain in clinical trials [42–49]. The BPI 3, or the worst pain rating, is recommended by the BPI developers as the primary response metric. BPI 3 indicates the worse pain over the prior 7 days on a numeric rating scale of 0–10, 0 being no pain and 10 being “pain as bad as you can imagine.” Additionally, the following other outcome measures will be collected: the daily least pain (BPI 4); average daily pain (BPI 5); daily present pain (BPI 6); and the after-morning ADL pain (BPI 9). The BPI 9 assesses how pain interferes with seven domains of daily activity. The domains assessed are general activity, mood, walking ability, normal work, relationships with other people, sleep, and enjoyment with life. The composite BPI 9 score is the average score of the domains. This specific outcome measure has been used in previous studies assessing intramuscular PNS [16, 17, 50–55].

The FIT-HaNSA objectively measures the participants’ capacity. Capacity is defined by the Word Health Organization as what an individual can do in an idealized environment [56]. The FIT-HaNSA is a timed test which measures the functional ability of the upper limb while completing multilevel tasks requiring grip and manipulation of the hand, elbow and shoulder reaching, sustained work overhead, and sustained positioning. This outcome measure has been validated and known to be reliable in assessing patients with shoulder pathology [57]. The Shoulder Pain and Disability Index (SPADI) is a self-administered questionnaire which assesses pain and function. The SF-12 is a quality-of-life measure that assesses physical functioning, role of limitation because of physical and emotional problems, bodily pain, social function, general mental health, vitality, and general health perception [58]. This outcome measure has been utilized previously to assess change in quality of life in other intramuscular PNS trials [18, 52, 59, 60].

Central sensitization will be measured using 3 Quantitative Sensory Testing (QST) techniques. First, primary and secondary hyperalgesia will be evaluated by mechanical pressure-pain thresholds that are measured with a hand-held Wagner Instruments FPIX Pain Test Algometer (Wagner Instruments, Greenwich, CT, USA) with a 0.875-cm^2^ round, rubber tip. Measurements are taken at the ipsilateral mid-belly of the deltoid, the contralateral mid-belly of the deltoid, and the mid-belly of the tibialis anterior on the contralateral limb. The pressure-pain threshold is the point at which the perception of applied pressure changes to a perception of discomfort and is measured in kg/cm^2^. At each site, three readings are sampled and averaged. This analysis has been utilized to evaluate the function of Type III and IV afferent fibers in a vast number of pain syndromes as well as shoulder impingement syndrome to assess deep somatic tissue sensitivity [28–31, 61–74]. Pinprick pain threshold will be measured at the same three sites using a weighted pinprick stimulus that exerts a force of 8, 16, 32, 64, 128, 256, and 512 mN (PinPrick Stimulators, MRC Systems GmbH, Heidelberg, Germany) [75, 76]. The stimulators will be applied in ascending order at a rate of 2 s on and 2 s off until the participant reports a perception of sharpness. This will be completed in five trials. The average measurement of the first perception of sharpness for the five trials will be recorded as the final threshold. This measurement provides information on the function of A-fibers and has been utilized in a wide variety of studies for central sensitization including shoulder impingement syndrome [29, 77–81]. Central integration will be evaluated through measurement of the wind-up ratio. The wind-up ratio is the perceived magnitude of pain from a single pinprick stimulus compared to a train of 10 pinprick stimuli of the same force applied one per second. A force of either 256 mN or 512 mN will be used, with the optimal choice being the lowest force that produces a sensation of pain on the dorsum of the hand. Testing will occur at the same three anatomic testing sites. The mean pain rating of the train of stimulation will be divided by the mean pain rating of the single stimulus to calculate the wind-up ratio.

### Interventions

**Fig. 1:**
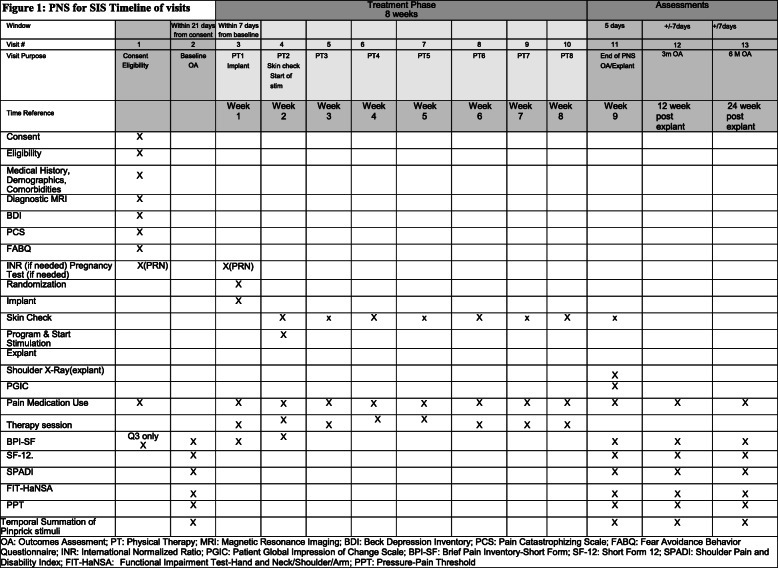
Peripheral nerve stimulation for shoulder impingement syndrome timeline of visits

Participants will be randomized to two treatment groups. The intervention group will be treated with active PNS of the axillary nerve of the affected shoulder and the control group will be treated with sham PNS of the axillary nerve of the affected shoulder. Both groups will receive a standardized exercise therapy program directed by a licensed therapist. Figure 1 is a table of the timeline of visits for this trial.

#### Peripheral nerve stimulation

The SPRINT endura PNS System (SPR Therapeutics, Cleveland, OH, USA) is used to deliver the PNS. The system consists of a small external stimulator and mounting pad, a percutaneous IM lead, and a hand-held remote control. The external stimulator is powered with rechargeable battery packs. The single-channel stimulator outputs a biphasic current waveform with current pulse parameter ranges that are suitable for PNS. The electrode has a coiled helical configuration wound from a seven-strand type-316LVM stainless-steel wire insulated with a poly-fluorocarbon. These electrodes have been used extensively to deliver percutaneous PNS to shoulder muscles [16, 17, 26, 51, 52, 59, 60, 82, 83].

Placement of the electrical lead is performed under a sterile condition. The location and depth of the electrode implant site are determined by monopolar needle stimulation. The monopolar needle is housed within an external introducer sheath and is inserted perpendicularly to the skin surface and advanced to the depth (3–4 cm) with demonstration of strong contraction of both the middle and posterior deltoid muscles [23, 59, 60, 83]. After demonstration of contraction, the external sheath is left in place and the percutaneous PNS lead is placed within the external sheath. After demonstration of muscle contraction, the introducer is then withdrawn with the electrode retained in the muscle by a barb at its tip. Both treatment groups have an electrode implanted by a study physician, though the control group does not have confirmatory electrical stimulation. The active group will receive active stimulation of a lead placed at the motor point between the middle and posterior deltoid. The other group will receive placebo stimulation of an electrode placed intramuscularly.

After 1 week of lead stabilization, both groups return to receive instructions on site care and the stimulator function. The treatment group receives a SPRINT endura programmed for stimulation to provide strong fused comfortable muscle contraction with minimal fatigue. Stimulation frequency (12 Hz) is fixed. The amplitude (0.2–30 mA) and pulse duration (10–200 μs) are set to produce strong, comfortable contraction of both the middle and posterior deltoids. A charge-balanced biphasic waveform allows an equal amount of charge to flow in either phase, creating a safe net zero charge [84]. Participants receive 6 h of stimulation per day, which can be delivered in single or divided sessions at the convenience of the user. The stimulator keeps an electronic log for compliance monitoring. Stimulation is delivered for 53 days.

The control group receives instructions and site care on stimulator function; however, the stimulator does not provide active stimulation. In all other respects, the stimulator appears to function in the same manner as the treatment group, including drainage of battery charge.

At the conclusion of the treatment phase, the electrode is removed by applying gentle traction to the external portion. All participants receive radiographic surveillance for retained electrode fragments, which are recorded as adverse events (AE).

#### Exercise therapy

Each participant, whether they are in the treatment group or the control group, receives formal ambulatory exercise therapy adapted from the Holmgren Protocol [85] once per week for 8 weeks. Each session is 30 min to 1 h, depending on participant ability to complete all exercises. The protocol consists of six exercises: two eccentric strengthening rotator cuff movements; three exercises for concentric/eccentric strengthening of scapular stabilizers; and posterior shoulder stretch. Progression of external load, with weight or elastic bands, varies with the participant’s tolerance. During each session, participants are trained in the implementation of the exercises, which are individually adjusted with increasing external loads by using weights and elastic rubber bands. Each strengthening exercise is repeated 15 times in three sets twice per day during the 8-week treatment period. The posterior shoulder stretch is performed for 30–60 s and is repeated three times, twice daily. Participants are advised that they are not to exceed a pain level of 5 on a pain numeric rating scale of 0–10 when performing the exercises. After completion of an exercise session, pain must return to the pre-session level before the next session; otherwise, the external load is decreased.

### Sample size calculation

Based on prior studies [23, 60], an average reduction of 5 points (63%, assuming a baseline of 8) in BPI 3 is anticipated for the treatment group by the end of treatment, with maintenance of this effect through the follow-up period. Based on our prior RCT of PNS for hemiplegic shoulder pain [33], a 2.5-point (31%) reduction is anticipated by the treatment of PT for the control group. The anticipated difference of 2.5 points between groups at 24 weeks exceeds the minimum clinically important difference in pain scores [86, 87]. To detect this difference with 80% power, α = 0.05, and anticipated SD of 2.9, the minimum sample size is 46 per group. With the anticipated drop-out rate of 20%, a sample size of 58 per group for a total of 116 participants is required.

### Statistical analysis

The primary analysis will be an intention-to-treat analysis with a comparison of treatment groups that includes all participants as originally allocated after randomization. Before linear mixed models are fitted, unadjusted exploratory looks at the data across the two groups will be completed. Plots of both the individual levels and group means over time by treatment arm are used to describe and inspect outcome trajectories. The outcome variables that will be analyzed in this manner include the BPI 3, BPI 4, BPI 5, BPI 6, BPI 9, SPADI, FitHANSA, and SF-12. A secondary aim is testing the hypotheses that pain reduction is associated with an increase of pain thresholds at the affected shoulder (primary hyperalgesia) and at the contralateral shoulder or tibialis anterior (secondary hyperalgesia), and the treatment group will have a greater increase than the control group. If pain reduction is associated with modulation of central sensitization, the trajectories of pain thresholds should parallel the anticipated trajectories for the 7-day highest BPI 3 score. If PNS mediates central sensitization to a greater extent than therapy, pain thresholds should increase in the treatment group relative to controls at the affected shoulder, which suggests reduction of primary hyperalgesia, and at the unaffected shoulder or unaffected tibialis anterior, which suggests reduction in secondary hyperalgesia; however, if either treatment group shows reduction of primary hyperalgesia in the absence of reduction of secondary hyperalgesia, it is unlikely that modulation of central sensitization is responsible. Rather, other mechanisms such as changes in biomechanics, peripheral sensitization, and other local physiology may be the mechanism.

We will evaluate whether pain reduction leads to a reduction in central sensitization using regression analyses. If a significant difference in pain reduction and central sensitization in the treatment group relative to the control group is demonstrated, and an association between pain reduction leads and a reduction in measures of central sensitization are demonstrated, a mediation analysis will be performed to test the hypothesis that the PNS leads to pain reduction relative to exercise therapy with structural equation modeling (SEM) [88, 89]. The SEM framework is flexible and allows construction of a statistical model corresponding precisely with the present study’s conceptual framework leading to clear hypothesis articulation [90]. The inclusion of three types of thresholds at three locations, in addition to including all one at a time in a structural model, multiple outcomes will be included in measuring central sensitization simultaneously in a modified model.

The third aim of this project is to determine whether a specific phenotype of participant characteristics will differentiate PNS responders from non-responders. Multiple measures in different domains are collected at baseline, including psychological traits (Pain Catastrophizing Scale [91], Fear Avoidance Beliefs Questionnaire [92, 93]), mood (Beck Depression Inventory-II [94]), demographics, sex, structural anatomy (imaging studies), and sensory testing (hyperalgesia, pain thresholds, and central integration). Cluster analyses are used to determine the baseline measures most predictive of treatment success in general and for each treatment. Appropriate cut-off values for these measures are estimated to use as guidelines for making treatment decisions. Multiple regression models are employed to predict the efficacy of each treatment for pain relief. Logistic regression models are used to confirm these results, with “responsive” and “non-responsive” groups based on meaningful change (30%) and substantial change (50%), as recommended for trials of chronic pain [95].

### Safety

The implantation of the lead confers minimal risk to the participants, with the probability of serious AE of 0.0006 per electrode implant [17]. There is minimal pain from the implantation procedure. The risk of pain is limited by the utilization of local anesthetic. The risk of syncope is limited by completing the procedure in a supine position for participants who are known to have a risk of syncope. Infection risk is limited by completing the implantation with sterile technique. The participants will be educated on appropriate care and cleansing of the region of implantation. Lead migration is possible, and if the lead is completely removed unintentionally, the participant will be asked if they are agreeable to have another lead implanted. A common risk of skin irritation can occur. It this occurs, the medical grade adhesive will be moved to a location of healthy skin. The medical grade adhesive will never be placed over an area of unhealthy skin. If the stimulation is to painful, the participant is instructed to turn off the stimulator. To assess for lead fracture, a post-explantation X-ray will be completed. This risk is uncommon. The risk of pain increasing with therapeutic exercise can occur. This risk is limited by utilizing highly trained therapists.

### Data management

All study documents and source documents are stored and maintained in a participant file. These documents include informed consent, laboratory results, radiology reports, copies of medical record, paper questionnaires, and other patient identifiable material. Each study candidate and participant will be assigned a study identification number in that is associated with study data in the electronic data system. A separate, secure database at each site will store a key linking the identifier with protected health information of the participant. All study data will be entered directly into an electronic data-entry system (REDCap) that is secure and compliant with regulations (21 CFR Part 111, FISMA, and HIPPAA-compliant environments). The investigator team will have access to the final dataset.

### Monitoring

A data and safety monitoring board ensures the safety of the participants and scientific validity of the study. This board consists of five members, including two physicians, three PhD scientists, and one PhD psychologist. The data and safety monitoring board is an independent board, free from influence of the sponsor and free of competing interests. The board reviews data, evaluates AEs, monitors the integrity of the data, and assures the conduct of the study is acceptable. Any recommendations of the data and safety monitoring board will be directly made to the principle investigator. There are no futility or efficacy stopping rules. The trial will be stopped if the data and safety monitoring board identifies a significant safety concern which would warrant stopping the trial. Any protocol modifications will come directly from the primary investigator.

In addition to real-time monitoring by the principal investigator, the lead site convenes quarterly meetings to review AEs. All AEs will be reviewed and a consensus is made for classification of the relatedness, whether the event was expected or unexpected and if the event was serious or not.

## Discussion

This study protocol will allow the investigators to determine if this novel, non-pharmacologic treatment of shoulder pain can demonstrate the same benefit in musculoskeletal patients which has been previously demonstrated in the stroke population.
