# Correction: The protocol for a multisite, double blind, randomized, placebo-controlled trial of axillary nerve stimulation for chronic shoulder pain

**DOI:** 10.1186/s13063-025-08955-y

**Published:** 2025-08-07

**Authors:** Travis Cleland, Nitin B. Jain, John Chae, Kristine M. Hansen, Terri Z. Hisel, Douglas D. Gunzler, Victoria C. Whitehair, Chong H. Kim, Richard D. Wilson

**Affiliations:** 1 MetroHealth Rehabilitation Institute, MetroHealth System, 4229 Pearl Rd, N5-27, Cleveland, OH 44109 USA; 2 Vanderbilt University Medical Center, 3319 West End Ave, Nashville, TN 37203 USA; 3 Center for Healthcare Research and Policy, MetroHealth System, 2500 MetroHealth Dr, Cleveland, OH 44109 USA

**Correction: Cleland et al. Trials 21, 248 (2020)**

**https@@@://doi.org/** 10.1186/s13063-020-4174-x

Following the publication of the original article [1], it was noticed that an incorrect description of the power analysis was incorporated into the manuscript and was published. The design of the study had a higher power to detect a smaller difference than was published. In accordance with NLM (https://www.nlm.nih.gov/bsd/policy/errata.html) and similar guidelines from the International Committee of Medical Journal Editors and the Committee on Publication Ethics, the authors are submitting this erratum to correct the error.

## Originally published power analysis:

Based on prior studies [23, 60], an average reduction of 5 points (63%, assuming a baseline of 8) in BPI 3 is anticipated for the treatment group by the end of treatment, with maintenance of this effect through the follow-up period. Based on our prior RCT of PNS for hemiplegic shoulder pain [33], a 2.5-point (31%) reduction is anticipated by the treatment of PT for the control group. The anticipated difference of 2.5 points between groups at 24 weeks exceeds the minimum clinically important difference in pain scores [86, 87]. To detect this difference with 80% power, α = 0.05, and anticipated SD of 2.9, the minimum sample size is 46 per group. With the anticipated drop-out rate of 20%, a sample size of 58 per group for a total of 116 participants is required.

## Revised power analysis (changes marked in bold):

Based on prior studies [23, 60], an average reduction of 5 points (63%, assuming a baseline of 8) in BPI 3 is anticipated for the treatment group by the end of treatment, with maintenance of this effect through the follow-up period. Based on our prior RCT of PNS for hemiplegic shoulder pain [33], a 2.5-point (31%) reduction is anticipated by the treatment of PT for the control group. **The study was powered to detect** the minimum clinically important difference of **2 points** in pain scores [86, 87]. To detect this difference with **9**0% power, α = 0.05, and anticipated SD of 2.9, the minimum sample size is 46 per group. With the anticipated drop-out rate of 20%, a sample size of 58 per group for a total of 116 participants is required.
